# Supplementary material for: New Insights on Molecular Autopsy in Sudden Death: A Systematic Review
Source: Diagnostics (Basel). 2024 May 30;14(11):1151. doi: 10.3390/diagnostics14111151 (PMC11171636; doi:10.3390/diagnostics14111151)
Supplement: Supplementary file 1 [file diagnostics-14-01151-s001.zip › Table S1.pdf]

Table S1. Table containing all data extrapolated from the studies considered.

Authors, date of publication and country	Study	Gene(s)	Heart disease	Sample characteristics	Sample number	Results	Observation
Zhen X. et al. (2023), China [11]	Case-control study	1 gene - $(CAG)_n$ repeat polymorphism within Androgen Receptor (AR) gene	Coronary heart disease	DNA was extracted from blood samples.	- 746 participants, comprising 182 cases of SCD-CAD (sudden cardiac death from coronary artery disease)<br>- 564 were healthy controls.	Individuals with shorter alleles (≤ 26 repetitions) exhibit a significantly reduced risk of SCD-CAD compared to those with longer alleles (OR = 0.343) in males, with a trend towards increased risk in males with shorter alleles. Finally, the study suggests that the role of different allele lengths may vary across various pathological conditions, drawing comparisons with results from other studies on (CAG)n polymorphisms.	The study highlighted an association between the (CAG)n polymorphism of the AR gene and the risk of SCD-CAD, with a sex- and allele length-dependent effect observed in male subjects. However, further research is needed to confirm these findings, particularly in larger samples of female subjects.
Neubauer J. et al. (2018), Switzerland [12]	Retrospective cohort study	393 cardio-vascular and metabolic disease genes have been investigated	Not specified	DNA was extracted from blood samples.	- 39 cases of sudden unexplained death were	Genetic testing disclosed three pathogenic and five likely pathogenic	This study demonstrated the importance of multidisciplinary

					examined. Of these 10 were left without a cause of death and underwent a molecular autopsy	variants in five out of the 10 SUD cases. In six out of these 10 families, no further phenotypically affected family members were discovered to date. In the remaining four families, clinical screening identified additional family members at risk. These findings had decisive consequences for the at-risk family members including life-long medication or implantation of an ICD for primary prevention, but these interventions might save lives.	collaboration in the management of cases of sudden unexplained death. It also allowed the identification of genetic variants associated with sudden cardiac death and permitted the identification of relatives at risk, thus helping to prevent further tragedies within the families involved.
Alhassani S. et al. (2018), Canada [13]	Research article	30 genes (AKAP9, ANK2, CACNA1C, CACNB2, CASQ2, CAV3, DSC2, DSG2, DSP, GPD1L, HCN4, JUP, KCNE1, KCNE2, KCNE3, KCNH2, KCNJ2, KCNJ5, KCNJ8, KCNQ1,	Not specified	Not specified	- A case of SUDY and his family	The analysis revealed a large deletion involving exons 4 to 14 of the PKP2 gene. The deletion was assumed to be pathogenic and was used for cascade	The observations serve to highlight that mutations associated with ventricular cardiomyopathy may alternately manifest with atrial phenotypes,

		NKX2.5, PKP2, RANGRF, RYR2, SCN1B, SCN3B, SCN4B, SCN5A, SNTA1, TMEM43)				screening to attempt to clarify its role in the sudden death of the proband and its potential impact on other family members. Although the living family members carrying the PKP2 deletion have yet to manifest definite clinical features associated with ARVC, the ventricular ectopy observed in the mother and younger brother may be reflective of an emerging phenotype.	potentially in isolation. They also serve to reinforce the notion that atrial cardiomyopathy may represent a genetic subphenotype of AF.
Alape Ariza J. et al. (2022), Colombia [14]	Case report (a 15-year-old who died in his sleep)	Clinical exome that includes 4834 clinically relevant genes for a suspected case of SCD	Cardiac channelopathy	DNA was extracted from a blood sample recovered during the autopsy		Five genetic variants were identified: C2992G, G1712A, C49424A, G275C, and G6955A present in the MYBPC3, KCND3, TTN, KCNH2, and ANK3 genes, respectively. All five rare potentially pathogenic exome variants were classified as poorly	These findings might indicate that the genetic variations identified could be potential factors associated with sudden cardiac death. Additionally, the homozygous variant in the KCNH2 gene associated with long QT syndrome type 2 seems to suggest the

						tolerated by at least two variant prediction algorithms. The variants found in the genes MYBPC3, TTN, and ACTN2 have been associated with hypertrophic cardiomyopathy, channelopathies, and other arrhythmias. If they are simultaneously presented, those pathogenic variants might be strong candidates to trigger sudden cardiac death.	patient's death was due to a cardiac channelopathy. These results recommend further molecular dynamics studies among these genetic variants and the tragic event.
Marey I. et al. (2020), France [15]	Retrospective study	15 genes (MYH7, MYBPC3, TNNT2, TNNI3, MYL2, PKP2, DSP, DSG2, LMNA, TTR and the five major sarcomeric)	Not specified	DNA was extracted from 17 frozen tissues, 6 post-mortem frozen blood, 1 post-mortem paraffin embedded tissue and 11 clinical samples (blood and fibroblasts)	35 patients with cardiac death and suspected cardiomyopathy based on autopsy or clinical data.	15 causal variants were identified in 15 patients (43% of the cohort): three causal variants in the DSP gene (desmoplakin), three in LMNA (lamin A/C), three in TNNT2 (troponin T), two in TNNI3 (troponin I), two in MYH7 (beta myosin heavy chain 7), one	The study confirms the feasibility and effectiveness of post-mortem genetic screening in patients with suspected cardiomyopathy. This type of analysis has been shown to be effective in detecting genetic variants associated with cardiomyopathies.

						in MYBPC3 (myosin-binding protein C) and one in TTR (transthyretin).	The genetic variants identified have had a significant impact on the diagnosis and management of the families involved, improving the clinical management of patients and their relatives. The study also highlights the complex ethical, legal and psychological issues associated with post-mortem genetic analyses. Furthermore, the importance of multidisciplinary teams and national coordination for the use of post-mortem genetic analyzes was highlighted.
Siskind T. et al. (2022), USA [16]	Retrospective Study	94 genes (ABCC9, ACTC1, ACTN2, AKAP9, AKAP10, ANK2, ANKRD1, ARHGAP24, BAG3, BCAT1, CACNA1C, CACNA2D1, CACNB2, CALM1, CAML2, CASQ2,	Not specified	Not specified	15 decedents, of which 6 (40%) had gross or histologic evidence of cardiac pathology on post-mortem examination,	All 15 decedents had positive post-mortem testing. Variants, including both pathogenic/likely pathogenic and variants of uncertain significance (VUS),	The diagnostic yield in this cohort was 22% which is within the range in the current literature. While this yield is similar to published reports, the population of this

		CAV1, CAV3, CDKN1A, CRYAB, CSRP3, CTF1, DES, DPP6, DSC2, DSG2, DSP, DTNA, EMD, FHL2, FLRT2, GATAD1, GLA, GPD1L, HAND1, HCN4, JUP, KCNA5, KCND3, KCNE1, KCNE1L, KCNE2, KCNE3, KCNE4, KCNH2, KCNJ2, KCNJ5, KCNJ8, KCNQ1, LAMA4, LAMP2, LDB3, LMNA, MYB, MYBPC3, MYH6, MYH7, MYL2, MYL3, MYLK2, MYOZ2, MYPN, NEBL, NEXN, NOS1AP, PC3, PKP2, PLN, PRKAG2, RANGRF, RBM20, RyR2, SCN10A, SCN1B, SCN2B, SCN3B, SCN4B, SCN5A, SGCD, SNTA1, STRN, TAZ, TCAP, TGFB3, TMEM43, TMPO, TNNC1, TNNI3, TNNT2, TPM, TPM1, TRPM4, TTR, VCL)			while the remaining nine had negative post-mortem examinations.	were identified in 13 genes, nine of which correlated with inherited arrhythmia syndromes (RYR2, SCN10A, KCNH2, CASQ2, TRDN, SCN5A, KNCA5, CACNA1C) and 5 with inherited cardiomyopathies (PRDM16, MYH7, MYBPC3, MYH6, MYLK2). Fifteen families and 32 first-degree relatives were referred for an evaluation. Familial variant-specific genetic analysis was performed on a total of 29 first-degree relatives. Fifteen were positive for the same variant identified in the decedents. In total, 43 decedent variants were tested, and 19 were found in first-degree relatives. One pathogenic (MYBPC3) and two likely pathogenic	study is more racially heterogeneous which may lead to discrepancy from other published data. Given the inability to fully predict the cause of SUD, these cases highlight the importance of familial evaluation even when post-mortem genetic testing is inconclusive. Though no phenotypes were directly linked to these findings, the screening of relatives led to medical treatment or device implantation in 3 individuals.

						(RYR2, MYH7) mutations were identified in relatives. A potential of heritable cause of sudden death was found in five of 15 families (33%) and seven of 32 family members (22%). The referral and evaluation of these individuals led to the diagnosis of seven potentially pre-symptomatic disease carriers and 2 clinically affected but genotype-negative individuals.	
Clemens D.J (2020), USA [17]	Epidemiological study	TRDN, which encodes for cardiac triadin	Triadine knockout syndrome (TKOS)	Not specified	857 sudden unexplained death cases. Of these, 599 were infant death syndrome (SIDS) and 258 were sudden unexpected death in the young (SUDY)	None of the 599 SIDS cases or 258 SUDY cases harbored either homozygous or compound heterozygous TRDN null variants. In fact, none of the SIDS or SUDY cases hosted even a single TRDN null allele. It was also assessed all cases for the presence of any	These findings indicate that, despite its malignant and potentially lethal phenotype, TKOS does not seem to contribute meaningfully to either SIDS or SUDY, at least among white decedents. The most likely explanation for the absence of TKOS in this sudden death

						novel homozygous or compound heterozygous missense variants which could potentially be disease causing, but none were identified.	cohorts is simply the extreme rarity of the disease. This study also suggests that TRDN is a difficult gene to sequence successfully. Two other potential explanations for this finding are age at death since and ethnicity.
Marzialiano N. et al. (2021), Italy [18]	Case report (a 32-year-old who died during physical activity and subsequently tested positive for SARS-CoV-2 infection)	60 genes (ACTC1, ACVRL1, APOB, BAG3, BMPR2, BRAF, CACNA1C, CASQ2, DES, DMD, DSC2, DSG2, DSP, ELN, EMD, ENG, FBN1, FLNC, GATA4, GLA, JAG1, JUP, KCNE1, KCNE2, KCNH2, KCNJ2, KCNJ8, KCNQ1, KRAS, LAMP2, LDLR, LDLRAP1, LMNA, MYBPC3, MYH7, MYL2, MYL3, NF1, NKX2-5, PKP2, PLN, PRKAG2, PCSK9, PTPN11, RAF1, RBM20, RYR2, SCN1B, SCN5A, SOS1, SOS2, TAZ,	Hypertrophic cardiomyopathy and heterozygous familial hypercholesterolemia	Not specified		During autopsy, several cardiac abnormalities were detected, including increased heart weight, thickening of the heart walls (hypertrophy), and diffuse nonobstructive coronary artery disease, indicative of pre-existing heart conditions. During the molecular autopsy two variants were identified: a heterozygous genetic variant in the MYH7 gene and a missense heterozygous mutation in the	The case described represents an example of a potential SARS-CoV-2 infection role in triggering or clinically unmasking inherited cardiovascular disease, whose combination might explain the cause of the patient's exitus. However, it is not possible to assess which was the exact cause of cardiac arrhythmogenic activity described in this report. It also highlights the importance of

		TGFBR2, TMEM43, TNNC1, TNNI3, TNNT2, TPM1, TTN, TTR)				LDLR gene, both classified as pathogenic. Indeed, these two mutations are already described as responsible for HCM and HeFH, respectively. Further analysis conducted within the family showed that LDLR mutation was maternally inherited, with 13 family members being carriers of the LDLR mutation, while MYH7 genetic lesion was de novo. All family member carriers of the LDLR mutation had systematic LDL plasma concentrations above >130mg/dL and positive records of cardiac and vascular ischemic events at young age. Six patients of this family, including the proband, died due to	autopsies and post-mortem genetic analysis to reveal the hidden pathogenetic mechanisms linked to SARS-CoV-2 infection. Overall this work strengthens knowledge for monitoring patients with congenital cardiovascular diseases, such as HCM and HeFH, revealing how SARS-CoV-2 infection could be harmful. The results of the molecular autopsy are of fundamental importance for first- or second-degree relatives in whom the identification of the pathogenic substrate, which makes them vulnerable to an increased risk of life-threatening cardiac events, including sudden death, could lead to clinical and

						cardio-vascular complications.	personalized treatments.
Beccacece L et al. (2023), Italy [19]	Research article	The DNA was genotyped for about 720,000 genetic markers	Not specified	DNA was extracted from blood samples	30 autopsy cases	More than 2000 variants were identified with statistically significant differences in the frequencies between the cases and controls, which might be associated with SCD. Among these variants, 356 SNPs, having the highest statistical values between the analyzed populations, were selected for further investigation. The top SNPs map inside or near 456 genes, both in coding and non-coding regions. The majority of these polymorphisms had not previously been implicated in any phenotype and disease; however, there were 25 variants that had shown a previous	Owing to the large number of variants and genes related to SCD that were discovered, this study supports that sudden cardiac death is a polygenic trait, in which the normal and altered activities of many genes contribute to the pathogenesis of cardiovascular conditions leading to death. Nevertheless, the current lack of involvement of many variants in cardiovascular diseases makes it necessary to investigate these polymorphisms further and more deeply, with the aim of clearly defining their roles in the pathogenesis of SCD and whether they will be useful as potential diagnostic

						association with cardiovascular diseases and phenotypes that increase the risk of developing these pathologies, confirming the hypothesis of this study. Among these variants, 25 SNPs could be considered to be likely pathogenic for SCD in the study, as they were consistent with previous publications showing an association with cardiovascular diseases or other risk factors for the development of these pathologies.	markers allowing for prevention measures.
Iglesia M. et al. (2021), Spain [20]	Retrospective study	The number of genes studied ranged from 194 to 380	Not specified	DNA was extract from blood samplesretrieved during the autopsy	31 cases of sudden death	Genetic testing was negative and labeled as wild type in 32.25% (n = 10). The remaining 67.74% (n = 21) were positive for some of the variants of interest: variants of unknown	In this study, the diagnostic yield rate of genetic testing was 67.74% including VUS, LPV and PV. The rate decreases to 6.45% if only considering PV and LPV, which

						significance (VUS) 61.29% (n = 19), likely pathogenic variants (LPV) 3.23% (n = 1), and pathogenic variants (PV) 3.23% (n = 1). The yield of molecular autopsy decreased to 6.45% when taking into account the pathogenic variants and likely pathogenic variants (n = 2). Genes involved were structural genes in 41.93% (n = 13) and related to channelopathies in 38.7% (n = 12). The molecular autopsies identified two different heterozygosis mutation in the MYBPC3 gene, previously described as pathogenic variants. In addition, were detected two variants of unknown significance, in the	indeed is a low diagnostic yield. Larger studies focusing on the evaluation of the utility of molecular autopsy in the population are needed to reach more robust results and to help to elucidate this issue. This is relevant for diagnostic purposes, and as guidance for familial screening to detect healthy carriers and affected ones. Clinical relevance of the VUS and their relation to disease is another relevant aspect that needs to be elucidated considering their frequent appearance in genetic test results.

						genes TPM1 and LDB3.	
Larsen M.K. et al. (2019), Denmark [21]	Retrospective study	104 genes (ABCC9f, ACTC1a,g, ACTN2l, AKAP9, ANK2, ANKRD1l, BAG3, CACNA1Cc, CACNA1D, CACNB2, CALM1h,n, CALM2h,n, CALM3h,n, CALR3, CASQ2, CAV3c, CRYAB, CSRP3a, CTF1, DESk, DMD, DPP6, DSC2, DSG2a, DSPa, DTNA, EYA4, FHL2, FKTN, GAAa, GJA5i,j, GLAl, GPD1Ld, HCN1e, HCN4e,f,g, ILK, JPH2f, JUP, KCNA5, KCND3f, KCNE1f, KCNE2f, KCNE3f, KCNE4f, KCNE5f, KCNH2f,o, KCNJ2f,o, KCNJ5f, KCNJ8f,h, KCNQ1e,h,o, LAMA4, LAMP2, LDB3g,l, lHCM, LMNAf,g,k, MOG1, MYBPC3a,g, MYH6a,e,	Hypertrophic cardiomyopathy (HCM), dilated cardiomyopathy (DCM), arrhythmogenic right ventricular cardiomyopathy (ARVC), long QT syndrome (LQTS), Familial atrial fibrillation (FAF)	DNA was extract from blood samples retrieved during the autopsy	70 cases of suspected sudden death	The cohort was divided into three groups: suspected cardiomyopathy, left ventricular hypertrophy and structural normal heart. In total, 11 individuals had pathogenic or likely pathogenic variants. In the hypertrophy group, the pathogenic variant was found in SCN5A, a gene associated with cardiac channelopathy. However, variants in SCN5A have also been found to be associated with DCM suggesting a causal relationship between variants in SCN5A and DCM. Only one individual with hypertrophy, though, had a pathogenic variant. The low number of	The study highlights the importance of genetic investigation in the context of sudden and unexpected deaths in young people. It was used a larger panel of 100 genes associated with inherited heart disease. This expansion has led to the identification of a greater number of genetic variants. The study identified several genetic mutations, some of which have been associated with specific heart diseases, such as cardiac hypertrophy (HCM), dilated cardiomyopathy (DCM), arrhythmogenic right ventricular cardiomyopathy (ARVC), and other arrhythmic

		MYH7a,g , MYL2, MYL3, MYLK2, MYOZ2, MYPNl, NEBL, NEXNl, NPPA, PKP2b, PLNk,l, PRDM16g, PRKAG2f,m, PSEN1, PSEN2, RANGRFb, RBM20, RPS7, RPSA, RYR2f,k, SCN1Bf,i, SCN2Bb, SCN3Bf, SCN4Bf, SCN5Aa,c,e,f,g,h,i,j,k , SDHA, SGCD, SLC22A5, SLC25A4, SNTA1, STARD3, TAZg, TCAPl, TGFB3, TMEM43, TMPO, TNNC1l, TNNI3l, TNNT2a,g, TPM1a,g, TRPM4i,j, TTNa,k , VCLa)				observed pathogenic and likely pathogenic variants in the hypertrophy group indicates that other genetic or epigenetic components may be involved in the development of hypertrophy and that simple hypertrophy is not necessarily a precursor of HCM.	conditions. Furthermore, co-segregation studies could not be performed in the current study.
Girolami F. et al. (2022), Italy [22]	Brief research report	174 genes (ABCC9, ABCG5, ABCG8, ACTA1, ACTA2, ACTC1, ACTN2, AKAP9, ALMS1, ANK2, ANKRD1, APOA4, APOA5, APOB, APOC2, APOE, BAG3, BRAF, CACNA1C, CACNA2D1, CACNB2, CALM1,	Not specified	Genomic DNA was extracted from blood samples	14 cases of sudden cardiac death (SCD)	In 12 out of 14 cases, the autopsy results were inconclusive; however, combining autopsy and genetic testing allowed a definitive diagnosis in a case. In the majority of cases, autoptic results were uncertain or inconclusive. Genetic	This study supports the idea that genetic test represents a significant source of information to infer the cause of death in otherwise healthy young people. Building of these promising observations, the use of more extended

		CALR3, CASQ2, CAV3, CBL, CBS, CETP, COL3A1, COL5A1, COL5A2, COX15, CREB3L3, CRELD1, CRYAB, CSRP3, CTF1, DES, DMD, DNAJC19, DOLK, DPP6, DSC2, DSG2, DSP, DTNA, EFEMP2, ELN, EMD, EYA4, FBN1, FBN2, FHL1, FHL2, FKRP, FKTN, FXN, GAA, GATAD1, GCKR, GJA5, GLA, GPD1L, GPIHBP1, HADHA, HCN4, HFE, HRAS, HSPB8, ILK, JAG1, JPH2, JUP, KCNA5, KCND3, KCNE1, KCNE2, KCNE3, KCNH2, KCNJ2, KCNJ5, KCNJ8, KCNQ1, KLF10, KRAS, LAMA2, LAMA4, LAMP2, LDB3, LDLR, LDLRAP1, LMF1, LMNA, LPL, LTBP2, MAP2K1, MAP2K2, MIB1, MURC, MYBPC3, MYH11, MYH6, MYH7, MYL2,				investigation identified likely pathogenic (LP) variants in deceased patients. Overall, 3LP variants (75%) were found in genes associated with hypertrophic cardiomyopathy.	genetic analyses in genotype-negative SCD victims (exome, whole genome), paralleled by targeted in vitro studies, may offer further insight into the substrates of juvenile arrhythmias.

		MYL3, MYLK, MYLK2, MYO6, MYOZ2, MYPN, NEXN, NKX25, NODAL, NPPA, NRAS, PCSK9, PDLIM3, PKP2, PLN, PRDM16, PRKAG2, PRKAR1A, PTPN11, RAF1, RANGRF, RBM20, RYR1, RYR2, SALL4, SCN1B, SCN2B, SCN3B, SCN4B, SCN5A, SCO2, SDHA, SEPN1, SGCB, SGCD, SHOC2, SLC25A4, SLC2A10, SMAD3, SMAD4, SNTA1, SOS1, SREBF2, TAZ, TBX20, TBX3, TBX5, TCAP, TGFB2, TGFB3, TGFBR1, TGFBR2, TMEM43, TMPO, TNNC1, TNNI3, TNNT2, TPM1, TRDN, TRIM63, TRPM4, TTN, TTR, TXNRD2, VCL, ZBTB17, ZHX3, ZIC3)					
Neubauer J. et al. (2021),	Retrospective study	244 genes (ABCC8, ABCC9, ACAD9, ACADM, ACADS,	Not specified	DNA was extracted from	45 cases of sudden	14 pathogenic or likely pathogenic single nucleotide	A large portion of SVs identified in this study were located

Switzerland [23]		ACADVL, ACTA2, ACTC1, ACTN2, ACVRL1, ADAMTS10, AGL, AKAP9, ALG10, ALMS1, ANK2, ANKRD1, ASCL1, ATP5F1E, BAG3, BDNF, BMPR1B, BMPR2, BRAF, CACNA1C, CACNA2D1, CACNB2, CALM1, CALM2, CALM3, CALR3, CAMK2G, CASQ2, CAV1, CAV3, CAVIN4, CBL, CDH2, CHRM2, CLCA2, COA5, COL3A1, COL5A1, COL5A2, COL6A1, COL6A2, CPT1A, CPT2, CRYAB, CSRP3, CTF1, CTGF, CTNNA3, DCHS1, DES, DLG1, DMD, DMPK, DNAJC19, DNM1L, DOLK, DPP6, DSC2, DSG2, DSP, DTNA, ECE1, EDN3, EFEMP2, ELN, EMD, ENG, ETFA, ETFB, ETFDH, EYA4, FBN1, FBN2, FGF12,		shock-frozen kidney tissue	unexplained death	variant (SNVs) were identified in 10 (22.2%) out of the 45 SUD cases. These variants were located in genes that are linked to cardiomyopathies (1 SUD case), ion channelopathies (2 SUD cases), connective tissue diseases and/or congenital malformation syndromes (3 SUD cases), and metabolic diseases (4 SUD cases). A total of 18 structural variants (SVs) were identified in 15 out of the 45 SUD cases, located in 17 different genes (ABCC9, CDH2, DMPK, DPP6, EFEMP2, FXN, GPD1L, KCNJ2, LAMA4, NOS1AP, PDLIM3, PDSS2, PPA2, PRKAG2, PRKG1, PTPN11, TRPM4).	in the intergenic or intronic regions of the target genes and thus were of unknown significance. The evaluation of variants in these noncoding regions has always been challenging as the knowledge about their contribution to electrophysiological dysfunction is still very limited. This study shows that structural variant in cardiac disease-associated genes might be involved in some SUD cases. However, the functional interpretation of pathogenic SVs is complex and genetic evidence should be used cautiously in molecular diagnosis.

		FHL1, FHL2, FHOD3, FKRP, FKTN, FLNA, FLNC, FXN, G6PC, GAA, GATA4, GATA5, GATA6, GATAD1, GDNF, GJA1, GJA5, GJD4, GK, GLA, GLB1, GLRA1, GPD1L, GUSB, HADH, HADHA, HADHB, HCN2, HCN4, HEY2, HFE, HMGCL, HMGCS2, HRAS, HTR2C, ILK, JPH2, JUP, KCNA5, KCND2, KCND3, KCNE1, KCNE2, KCNE3, KCNE5, KCNH2, KCNJ2, KCNJ5, KCNJ8, KCNK17, KCNQ1, KLF10, KRAS, LAMA4, LAMP2, LDB3, LMNA, LRP5, LRRC10, LZTR1, MAOA, MAP2K1, MAP2K2, MED12, MED23, MOG, MRPL3, MT-TI, MT-TL1, MYBPC3, MYH11, MYH6, MYH7, MYL2, MYL3, MYLK, MYLK2,					

		MYO6, MYOM1, MYOZ2, MYPN, NEBL, NEXN, NKX2-5, NOS1AP, NOTCH1, NPPA, NPPA, NRAS, PDLIM3, PDSS2, PHOX2B, PKP2, PLEKHM2, PLN, PPA2, PPP1R13L, PRDM16, PRKAG2, PRKG1, PSEN1, PSEN2, PTPN11, RAB3GAP1, RAF1, RANGRF, RBM20, RET, RYR2, SCN10A, SCN1B, SCN2B, SCN3B, SCN4B, SCN5A, SCO2, SDHA, SEMA3A, SGCD, SHOC2, SKI, SLC22A5, SLC25A10, SLC25A3, SLC37A4, SLC4A3, SLC6A4, SLMAP, SMAD3, SMAD9, SNTA1, SOS1, SYNE1, SYNE2, TAZ, TBX1, TBX20, TBX3, TBX5, TCAP, TGFB2, TGFB3, TGFBR1, TGFBR2, TMEM43, TMEM70, TMPO, TNNC1, TNNI3, TNNI3K,					

		TNNT2, TP63, TPM1, TRDN, TRIM63, TRPM4, TRPM7, TSFM, TSPYL1, TTN, TTR, TXNRD2, VCL, XK, ZNF365)					
Scheiper-Welling S. et al. (2022), Germany [24]	Retrospective study	93 genes with known cardiac associations	Arrhythmic heart disease	DNA was isolated from autopsy whole blood or tissue samples	56 unrelated sudden death cases in the young	Genetic testing revealed a total of 53 rare protein-altering variants (MAF < 0.2%) in 32 different genes out of the 93 genes investigated and associated with inherited arrhythmogenic disease. While 17 of the variants were identified in core genes, another 36 were detected in minor genes. Eleven cases comprised more than one rare variant. Applying the American College of Medical Genetics and Genomics (ACMG) guidelines, two rare variants were classified as pathogenic. Most of the variants found in	This study shows that genetic testing by itself might be fairly meaningless if a VUS is detected. There are currently no specific forensic guidelines on the management and interpretation of SUD cases where a VUS has been detected. Therefore, the recommendation is to work with experienced multidisciplinary teams to properly interpret the genetic results and to provide adequate family counselling.

						our study were missense variations, categorized as VUS. Further sub-classification revealed that 5 VUS may be potentially pathogenic. Twenty-two cases (39%) exhibited no sequence variations. In the seven SUD cases with a clinically actionable variant, the family was contacted to provide recommendations and options for further management.	
Fadoni J. et al. (2022), Portugal [25]	Observational, transversal, and retrospective study	40 genes (MYBPC3, MYH7, TNNI3, TNNT2, ACTC1, TPM1, MYL2, MYL3, MYH6, TNNC1, VCL, CAV3, MYLK2, JPH2, CSRP3, ANKRD1, DES, ACTN2, MYL4, NEXN, CRYAB, DSG2, HSPB1, HSPD1, MYO6, GPD1L, KCNE2, NME1, MYC, POMC, SCN5A, TP53,	Hypertrophic cardiomyopathy (HCM)	DNA was extracted from peripheral blood retrieved during the autopsy	16 unrelated SCD	A total of 104 variants were identified among the 16 samples analyzed. Using the ACMG guidelines to interpret pathogenicity, we filtered out 91 genetic variants, classified as benign or likely benign. The remaining variants included one	Molecular autopsies are reported to uncover a likely or plausible cause of death in 29–40% of SCD cases. In this study, a pathogenic or likely pathogenic variant was identified in six of the 16 SCD victims (37.5%) what suggests that the genes selected in the

		ACAD9, GAA, PRKAG2, LAMP2, NDUFS1, RAF1, SCO2, SCL25A4)				pathogenic (MYBPC3 c.2221delG), one likely pathogenic (MYH7 c.G2348T), one VUS suggesting pathogenicity (MYLK2 c.C808T), and 10 VUS (ACAD9c.G976A; ACTN2 c.C1330T; ACTN2 c.842delG; DSG2 c.370delT; KCNE2 c.T170C; MYH6 c.5803delA; MYH7 c.2304delG; MYO6 c.973delA; NEXN c.1201delA; and NME1 c.413delA). In total, a pathogenic or likely pathogenic variant was detected in six (37.5%) SCD victims, including one of the victims with family history of SCD, and one with antemortem diagnosis of HCM. A VUS suggesting pathogenicity was found in one (6.25%) victim, and 10 VUS	analysis are relevant in HCM investigations. The application of NGS techniques can improve the diagnostic yield as it offers the possibility to analyze a large number of genomic regions simultaneously, which would be too expensive and time-consuming if performed by traditional Sanger sequencing. Nevertheless, from the large amount of information provided by NGS arises a new challenge in the genetic investigations, which is the high number of hard-to-interpret VUS.

						were detected in 9 (56.25%) victims, including one victim with antemortem diagnosis. In six (37.5%) SCD victims, no pathogenic, likely pathogenic variant, nor VUS was identified.	
Martinez-Barrios E. et al (2023), Spain [26]	Retrospective study	113 genes (ABCC9, ACTA2, ACTC1, ACTN2, AKAP9, ANK2, BAG3, CACNA1C, CACNA1G, CACNA1H, CACNA1I, CACNB2, CASQ2, CAV3, CHRM2, COL3A1, CRYAB, CSRP3, CTF1, DES, DMD, DMPK, DSC2, DSG2, DSP, ECE1, EMD, EN1, EYA4, FBN1, FHL2, FKTN, GJA7, GLA, GPD1L, HCN1, HCN2, HCN4, ILK, JPH2, JUP, KCNA4, KCNA5, KCND2, KCND3, KCNE1, KCNE2, KCNE3, KCNH2, KCNJ2, KCNJ3, KCNJ5,	Not specified	Not specified	51 post-mortem cases of sudden unexpected death in young population (<17 years)	In the group of sudden deaths in the first year of life no diagnostic changes were made. Reinterpretation of the data highlighted that some rare genetic variants previously classified as VUS have seen their harmful role downgraded to probably benign or benign due to an increase in frequency in the population. No variants were promoted to likely pathogenic or disease-causing. These changes were observed mainly due to the increase in	The study highlights the importance of periodic re-examination of rare genetic variants previously classified as VUS (variants of uncertain significance) in cases of sudden and unexplained deaths in young people. This review is crucial to identify the cause of sudden deaths and to offer more accurate genetic counseling to relatives, especially if they carry the same genetic mutation. The analysis showed that more than 10% of rare variants

		KCNK4, KCNQ1, LAMA4, LAMP2, LDB3, LMNA, MYBPC3, MYH6, MYH7, MYL2, MYL3, MYLK2, MYOZ2, MYPN, NEBL, NEXN, NOS1AP, NOTCH1, NPPA, NUP155, PDLIM3, PHOX2A, PHOX2B, PKP2, PLN, PRKAG2, PSEN1, PSEN2, RBM20, RET, RYR2, SCN10A, SCN1B, SCN2B, SCN3B, SCN4B, SCN5A, SGCA, SGCB, SGCD, SIRT3, SLC25A4, SLC6A4, SLC8A1, SLMAP, SNTA1, TAZ, TCAP, TGFB3, TGFBR1, TGFBR2, TLX3, TMEM43, TMPO, TNNC1, TNNI3, TNNT2, TPM1, TTN, and VCL)				allele frequency in the population. In the SIDS (sudden infant death syndrome) subgroup, due to rare VUS variants were also downgraded to benign due to the increase in allele frequency. Overall, reductions in allele frequency were observed compared to a 2017 study. In the VUS group, many variants were reclassified as VUS-LB or VUS-LP based on genetic category. In the group of young deceased suddenly excluding SIDS cases, some rare previously VUS variants were downgraded to potentially benign, mainly due to an increase in allele frequency in the population. No promotions to	previously classified as VUS had their potential harmful role downgraded due to an increase in allele frequency in the population. Furthermore, the article highlights that the main tendency for VUS variants to change is for variants that may have a more deleterious role (VUS-LP) and are often associated with genes encoding cardiac ion channels or associated proteins. In contrast, the majority of VUS variants with a tendency towards lower pathogenicity (VUS-LB) are associated with genes encoding myocyte structural proteins. However, the interpretation of genetic results should always be supported by clinical, familial and

						potentially pathogenic or pathogenic were observed. Similar to the global group, a decline in allele frequency was observed compared to 2017. In the VUS group, some variants were reclassified as VUS-LB or VUS-LP based on the genetic category. In summary, reinterpretation of the data highlighted changes in genetic variant classifications, but no variants were promoted to a more pathogenic category.	forensic data for accurate translation into clinical practice.
Tuveng Jon M. et al.(2018), Norway [27]	Case report	5 genes (KCNQ1, KCNH2, SCN5A, KCNE1, KCNE2)	Not specified	Not specified	A case of SUDY	The index patient was heterozygous for a novel KCNH2 mutation (c.185G > A, ref. seq.: NM_000238.2) in exon 2 predicting substitution of arginine at position 62 with	Lethal cardiac arrhythmias occurred in the woman who carried of a novel KCNH2 mutation with functional properties consistent with a loss-of-function and type 2 LQTS.

						glutamine (designated R62Q) in the encoded HERG potassium channel.	
Kraoua L. et al. (2012), Tunisia [28]	Research article	Whole genome	Hypertrophic/dilatated cardiomyopathy	DNA was extracted from blood samples	A case of SUDY	The analysis revealed a variant in the ACTN2 gene (c.355G > A; p.(Ala119Thr))	The current study highlights the utility of postmortem whole genome sequencing in the identification of causative gene variant underlying SCD and points out the importance of clinical examination which allowed the researchers to suspect a syndromic HCM occurring in the same family
Gélinas R. et al. (2019), Germany [29]	Case report (a 26-year-old Chinese woman who died in her sleep)	184 genes (AARS2, ABCC6, ABCC9, ACAD9, ACADVL, ACTA1, ACTA2, ACTC1, ACTN2, AGK, AGL, AKAP9, ALMS1, ALPK3, ANK2, ANO5, APOA1, BAG3, BRAF, CACNA1C, CACNB2, CALM1, CALM2, CALM3, CALR3, CAPN3,	Not specified	DNA was extracted from frozen heart tissue		The genetic testing revealed a homozygous missense variant in SLC22A5 gene. This variant was classified as pathogenic for PCD by the American College of Medical Genetics Standards and Guidelines. This is the first case of PCD	This report strengthens the link between metabolic disorders and arrhythmias and broadens the discussion regarding genetic testing in SCD and gene-elusive inherited arrhythmia syndromes. Metabolic

		CASQ2, CAV3, CBL, CDH2, COX15, CPT2, CRYAB, CSRP3, CTNNA3, DBH, DES, DMD, DNAJC19, DOLK, DSC2, DSG2, DSP, DTNA, DYSF, EEF1A2, ELAC2, EMD, ENPP1, EPG5, ETFA, ETFB, ETFDH, FBXO32, FHL1, FKRP, FKTN, FLNC, FOXD4, FOXRED1, FXN, GAA, GATA5, GATA6, GATAD1, GBE1, GFM1, GLA, GLB1, GMPPB, GTPBP3, GUSB, HADHA, HAND1, HCN4, HFE, HRAS, ISPD, JPH2, JUP, KCNA5, KCNE1, KCNE2, KCNH2, KCNJ2, KCNJ5, KCNQ1, KRAS, LAMA2, LAMP2, LARGE, LDB3, LMNA, LRRC10, LZTR1, MAP2K1, MAP2K2, MLYCD, MTO1, MYBPC3, MYBPHL, MYH6, MYH7, MYL2, MYL3, MYL4, MYOT,				presenting with SCD during adulthood in the absence of other cardiac manifestations. Caution should be exercised, however, when implicating SLC22A5 in cases of shortened QT interval or SCD because other determinants must also be considered.	investigation may therefore be considered in the presence of unexplained atrial and ventricular arrhythmias and cardiomyopathies and may provide an opportunity for disease-modifying therapy.

		MYPN, NDUFAF2, NEXN, NF1, NKX2-5, NOS1AP, NRAS, NUP155, PCCA, PCCB, PKP2, PLEC, PLEKHM2, PLN, PNPLA2, POMT1, PPA2, PPP1CB, PRDM16, PRKAG2, PTPN11, RAF1, RASA2, RBCK1, RBM20, RIT1, RMND1, RRAS, RYR2, SALL4, SCN10A, SCN1B, SCN3B, SCN5A, SCNN1B, SCNN1G, SCO2, SDHA, SELENON, SGCA, SGCB, SGCD, SGCG, SHOC2, SLC22A5, SLC25A20, SLC25A4, SMCHD1, SOS1, SOS2, SPEG, SPRED1, TAB2, TAZ, TBX20, TBX5, TCAP, TECRL, TGFB3, TMEM43, TMEM70, TNNC1, TNNI3, TNNI3K, TNNT2, TOR1AIP1, TPM1, TRDN, TRIM32, TRPM4, TSFM, TTN, TTR,					

		VCL, VCP, VPS13A, XK)					
Takahashi Y. et al (2023), Japan [30]	Research article	72 genes (ABCC9, ACTC1, ACTN2, AKAP9, ANK2, CACNA1C, CACNA2D1, CACNB2, CALM1, CALM2, CASQ2, CAV3, CSRP3, DES, DPP6, DSC2, DSG2, DSP, GJA5, GPD1L, HCN4, HEY2, IRX3, JUP, KCNA5, KCND3, KCNE1, KCNE2, KCNE3, KCNE5, KCNH2, KCNJ2, KCNJ3,CNJ5, KCNJ8, KCNQ1, LDB3, LMNA, MYBPC3, MYH6, MYH7, MYL2, MYL3, MYL4, MYOZ2, NEXN, PKP2, PLN, RANGRF, RBM20, RYR2, SCN10A, SCN1B, SCN3B, SCN4B, SCN5A, SGCD, SNTA, TAZ, TBX5, TCAP, TGFB3, TMEM43, TNNC1, TNNI3, TNNT2, TPM1, TRDN, TRPM4, TTN, TTR)	Not specified	DNA was extracted from blood and formalin fixed, paraffine embedded tissues.	17 cases of SCD	Several candidate variants were found in each case; the average number of candidates per case was 4.53 (range: 2 to 8). The same variants were rarely shared among the cases, and no variants were found at the splice sites. In addition, 42.9% of those variants were found in the titin (TTN) gene which encodes various sizes of TTN protein via alternative splicing, making it quite difficult to predict the clinical significance of the variants.	This study combined the information of clinical history, autopsy findings, and genetic analysis with or without family study in 17 cases where lethal arrythmia was suspected as the cause of death, and identified a nonsense variant in PKP2 and frameshift variant in TRPM4 in two cases of ACM. Some missense variants, previously regarded as VUS in the pathogenesis of ACM, were also identified in the two cases. On the other hand, in the remaining 15 cases, we could not find apparent associations between missense variants and the phenotype because no significant

							morphological changes were confirmed in the heart.
Yamamoto T. et al. (2019), Japan [31]	Case report (a male patient in his 20s)	Clinical exome	Myotonic dystrophy type 1 (DM1)	Genomic DNA was isolated from blood leukocytes. Total RNA was isolated from cardiac tissue and biceps muscle of the patient		The total number of detected variants was 207, including 3 frameshift variants, 1 stop-gained variant, and 167 missense variants. Among 167 missense variants, 28 were classified as deleterious and damaging in in silico algorithms. Only two variants are associated with myopathy and cardiac disease. One was Ile536Thr-RBM20, and the other was Val462Ala-DSC2. However, RNA-seq analysis shows abnormal alternative splicing of LDB3 in the patient samples compared with the control samples, which has been detected in skeletal muscle in DM1	RNA-seq analysis is useful to determine the exact molecular diagnosis for sudden cardiac death since exome sequencing cannot detect splicing abnormality. This study advocates performing RNA-seq analysis in cases of sudden cardiac death. It appeared that LDB3 alternative splicing due to expanded CTG repeats in DMPK was involved in cardiac muscle and skeletal muscle in a sudden cardiac death patient. RNA-seq analysis evoked the patient was DM1, but exome sequencing would have been misleading that RBM20 variant was causative. It was

						patients. These results lead the diagnosis that the patient is DM1 with expanded CTG repeats in DMPK. This finding suggests that one of the molecular mechanisms of sudden cardiac death in the asymptomatic subclinical DM1 patient might be LDB3 abnormal splicing due to the CTG repeat in DMPK.	determined that I536T-RBM20 was not the cause of phenotype in this patient.
Ripoll-Vera T. et al. (2020), Spain [47]	Research article	Not specified	Not specified	Genomic DNA was isolated from blood	62 cases of SCD	In 40 individuals, ACMG –AMP criteria classified the variants as VUS, pathogenic variants (PV), or likely pathogenic variants (LPV), representing a diagnostic yield of 64.5%. Considering only P and LP variants, the diagnostic yield was 30.6%. VUS, PV, or LPV were detected in	NGS is a very useful tool for the study of SCD, especially for follow-up family screening. This new tool has replaced traditional molecular autopsy, which examined a handful of genes by Sanger sequencing, most prominently KCNQ1, KCNH2, SCN5A, and RyR214,22. The

						samples from 21 individuals (70%). Of the detected variants, 15 were linked to channelopathies (10 to long QT syndrome, 3 to Brugada syndrome, and 2 to catecholaminergic polymorphic ventricular tachycardia) and 10 to structural genes (table 1). Considering only P and LP variants, the diagnostic yield was 26.6%.	diagnostic yield of the previous approach was 0-35%.23,24 By comparison, NGS successfully identifies genetic variants in 30% to 35% of individuals dying between the ages of 1 and 35 years and diagnosed with SUDS in the clinical autopsy.15,16,25 NGS offers many advantages over traditional genetic analysis, particularly in comparison with the high probability of false negatives and false positives with exome analysis, which results in high numbers of hard-to-interpret VUS (as many as 13 per deceased individual).
Grassi S. et al. (2021), Italy and Spain [32]	Case report	82 genes (ABCC9, ACTC1, ACTN2, AKAP9, ANK2, BAG3, CACNA1C,	Not specified	DNA was extracted from post-mortem whole blood	A case of SUDY	Applying current criteria for the assessment of the significance of the	Currently, postmortem genetic testing is not mandatory, but in

		CACNA2D1, CACNB2, CASQ2, CAV3, CRYAB, CSRP3, DES, DMD, DMPK, DSC2, DSG2, DSP, EMD, FKTN, FLNC, GLA, GPD1L, HCN4, JPH2, JUP, KCND3, KCNE1, KCNE2, KCNE3, KCNE5, KCNH2, KCNJ2, KCNJ5, KCNJ8, KCNQ1, LAMP2, LDB3, LMNA, MYBPC3, MYH6, MYH7, MYL2, MYL3, MYOZ2, MYPN, NEBL, NEXN, NOS1AP, PDLIM3, PKP2, PLN, PRKAG2, RANGRF, RBM20, RYR2, SCN1B, SCN2B, SCN3B, SCN4B, SCN5A, SCN10A, SGCD, SLMAP, SNTA1, TAZ, TCAP, TGFB3, TMEM43, TMPO, TNNC1, TNNI3, TNNT2, TP63, TPM1, TRDN, TRIM63, TRPM4, TTN, TTR, VCL)				variants, the significance of variant of TTN gene was interpreted as likely benign, while the variant of SLMAP was classified as of unknown significance. The analysis of other family members found that the variant was transmitted by his mother. Both the relatives reported no previous cases of SD or cases of arrhythmogenic syndromes in their families, were visited by a cardiologist and underwent resting 12-lead ECG and echocardiography, which were negative. In the light of this data, it is confirmed the unknown significance of the variant.	the cases of suddenly died young persons it can often give a substantial contribution to find the cause of the death

Modena M. et al. (2019), Italy [33]	Case report	Whole exome sequencing	Not specified	DNA was extracted from formalin fixed paraffine embedded left ventricular tissue	A case of SCD	Eleven variants were predicted as pathogenic: two had not been previously described, six had been previously described in the ClinVar Database and three single nucleotide variations without a clear clinical significance	WES and bioinformatic analysis allow the screening of the whole coding region of the genome in search for causative genetic mutations for sudden death and can be effectively used for the reassessment of unsolved cases of SUD. This molecular autopsy approach can either identify rarer pathogenic variants which can be missed by traditional sequencing techniques or provide novel candidate genetic variants to be tested in functional studies in order to shed new light into the pathophysiology of sudden death.
Shanks GW. et al. (2018), United	Research Article	99 sudden death-susceptibility genes	Not specified	DNA was isolated from autopsy whole	25 cases of unrelated sudden unexplained	27 ultrarare (MAF <0.00005) NSVs within the 99 sudden death-susceptibility	The study found that 64% of cases of sudden death in young people

Kingdom [34]				blood or frozen tissue	death in the young (SUDY)	genes in 16/25 (64%) individuals overall, including 9/12 (75%) black and 7/13 (54%) white decedents. Of the 27 NSVs identified, 4 were present in major genes for cardiac channelopathies and cardiomyopathies (KCNQ1, KCNH2, SCN5A, RYR2, MYH7, and MYBPC3) and 23 variants were in "minor" genes. None of the variants was identified within any of the 3 sudden unexplained death in epilepsy-susceptibility genes (KCNA1, SCN1A, and SCN8A). Of the 27 ultrarare NSVs, 10 NSVs (37%) were classified as pathogenic variants or likely pathogenic variants based on the ACMG guideline criteria. Overall, 4 of 25 SUDY cases (16%)	(SUDY) had ultra-rare genetic variants in genes associated with susceptibility to inherited heart conditions. This suggests that genetic variants could play a significant role in some cases of SUDY. It turned out that ultra-rare genetic variants were more common in cases of black individuals than white ones. This observation indicates the possibility of genetic differences between ethnic groups in susceptibility to sudden death. The study's findings highlight the importance of further research to confirm the association between genetic variants and sudden death in young people, as well as to evaluate their clinical

						harbored clinically actionable variants associated with severe cardiac pathologies such as Brugada syndrome and cardiac hypertrophy. However, some variants were identified, although they met the ACMG criteria, they were not consistent with the autopsy findings.	relevance more thoroughly. It was highlighted that despite the identification of genetic variants, their association with the cause of death should be carefully evaluated in combination with autopsy findings and family history to ensure adequate clinical management of surviving relatives.
Marcondes L. et al. (2018), New Zealand [35]	Retrospective study	Not specified, but the following genes are mentioned: SCN5A, KCNH2 KCNQ1, KCNE2, KCNE1 and KCNJ2	LQTS	Not specified	365 cases of unrelated sudden unexplained death in the young (SUDY)	In 21% of cases subjected to genetic analysis (27 out of 128), a total of 31 genetic variants were identified. Of these, 12% of males and 36% of females had a positive genetic variant (with significant gender differences). In 13 of 27 cases with genetic variants (48%), the variants were classified as "pathogenic", which	The study highlighted that 21% of cases of sudden death in young people (SUDY) showed genetic variants in some genes associated with hereditary cardiac arrhythmias. This highlights the importance of genetic analysis in evaluating the causes of sudden death in young individuals. Of the genetic

						represents 10% of all SUDY cases. These were called "final gene-positives". In 14 cases with genetic variants, it was not yet possible to determine whether the variant and the resulting channel dysfunction were the cause of death. Significant differences in demographic characteristics were observed between the gene-positive and gene-negative patient groups. For example, the majority of gene-positive patients were of European New Zealand (Caucasian) descent. Most gene-positive patients were aged between 25 and 40 years, but the probability of being gene-positive was higher in patients aged between 1 and	variants identified, 48% were classified as pathogenic. These variants are thought to be responsible for death in the SUDY cases in which they have been found. It is important to recognize these variations as they can be indicative of life-threatening inherited conditions. Some genetic variants initially classified as pathogenic were later reclassified as variants of uncertain significance or polymorphisms, meaning that they alone cannot be considered the cause of sudden death. This highlights the complexity of interpreting genetic variants and the importance of accurate analysis. Genetic analysis can reveal whether a

						12 years, particularly in the "final gene-positives" group	sudden death is linked to an inherited condition. This information is valuable for the prevention and management of inherited heart conditions in family members of deceased patients.
Jenewein T. et al. (2018), Germany [36]	Case report (a 17-year-old female)	13 genes (DSC2, DSG2, DSP, HCN4, KCNJ2, KCNQ1, KCNH2, SCN5A, KCNE1, KCNE2, PKP2, RyR2, SCN4B)	Not specified	DNA was extracted from blood samples		The heterozygous variant A572D as well as the polymorphism H558R in the SCN5A gene were found. The variant A572D has a MAF of 0.37% in the European population and has been found to be associated with LQTS.	The study found the presence of genetic variants in the SCN5A gene, which is known to be associated with LQTS and may influence the risk of serious cardiac events. The study does not provide detailed information on the relationship between these specific genetic variants and clinical symptoms, such as QT interval prolongation and risk of sudden cardiac death. However, it suggests that these variants

							may contribute in different ways to LQTS complexity and clinical manifestations.
Neubauer J. et al. (2018), Switzerland [37]	Observational study	189 genes (ABCC8, ABCC9, ACAD9, ACADM, ACADS, ACADVL, ACTA2, ACTC1, ACTN2, ACVRL1, ADAMTS10, AGL AKAP9, ANK2, ANKRD1, ASCL1, ATP5E, BAG3, BMPR1B, BMPR2, BRAF, CACNA1C, CACNA2D1, CACNB2, CALM1, CALM2, CALM3, CALR3, CAMK2G, CASQ2, CAV1, CAV3, CBL, COA5, COL3A1, COL5A1, COL5A2, CPT1A, CPT2, CRYAB, CSRP3, CTF1, CTGF, CTNNA3, DCHS1, DES, DLG1, DMD, DMPK, DNAJC, DNM1L, DOLK, DPP6, DSC2, DSG2, DSP, DTNA, ECE1, EFEMP2, ELN, EMD,	Not specified	DNA was extracted from shock frozen heart and kidney tissue in 31 SCD case. The DNA of the remaining three individuals was extracted from blood samples.	34 cases of unrelated sudden unexplained death	The study identified genetic variants with potential pathogenic effects in 10 out of 34 cases of sudden unexplained death, representing 29.4% of the cases examined. Genetic variants have been identified in various genes associated with several medical conditions, including Brugada syndrome (BrS), polymorphic catecholaminergic ventricular tachycardia (CPVT), pulmonary arterial hypertension (PAH), atrial fibrillation (Afib), long QT syndrome (LQTS), and others. The genetic variants identified included missense variants and other types of	The study detected genetic variants with potential pathogenic effects in a significant percentage of cases. This suggests that genetic variants could play a relevant role in the causes of sudden unexplained deaths. Some of the variants identified were associated with genes known to be linked to specific heart diseases and medical conditions. This suggests that the genetic variants identified could have an impact on susceptibility to these pathologies. Some of the identified genetic variants have not been previously described in genetic

		ENG, ETFA, ETFB, ETFDH, EYA4, FBN1, FBN2, FHL2, FKRP, FKTN, FLNA, FOXRED1, G6PC, GAA, GATA4, GATA5, GATA6, GATA6, GATAD1, GJA1, GJA5, GJD4, GK, GLA, GLB1, GPD1L, GUSB, HADH, HADHA, HADHB, HCN2, HCN4, HEY2, HFE, HMGCL, HMGCS2, ILK, JPH2, JUP, KCNA5, KCND3, KCNE1, KCNE1L, KCNE2, KCNE3, KCNE5, KCNH2, KCNJ2, KCNJ5, KCNJ8, KCNQ1, LAMA4, LAMP2, LDB3, LMNA, MAP2K1, MAP2K2, MED23, MRPL3, MYBPC3, MYH11, MYH6, MYH7, MYL2, MYL3, MYLK, MYLK2, MYOM1, MYOZ2, MYPN, NEBL, NEXN, Nkx2-5, NOS1AP, NOTCH1, PDLIM3,				potentially pathogenic genetic mutations. Some of the identified variants had not previously been described in genetic databases, while others were known and associated with specific medical conditions.	databases, suggesting that they may be rare variants or specific to this cohort. The study highlights the importance of genetic analysis, particularly whole exome testing (WES), in trying to identify possible genetic causes of sudden unexplained deaths.

		PKP2, PLN, PRKAG2, PRKG1, PSEN1, PSEN2, RAF1, RANGRF, RBM20, RYR2, SCN10A, SCN1B, SCN2B, SCN3B, SCN4B, SCN5A, SCO2, SDHA, SEMA3A, SGCD, SLC22A5, SLC25A3, SLC37A4, SLMAP, SMAD3, SMAD9, SNTA1, SYNE1, SYNE2, TAZ, TBX5, TCAP, TGFβ2, TGFβ3, TGFβR1, TGFβR2, TMEM43, TMPO, TNNC1, TNNI3, TNNT2, TPM1, TRDN, TRPM4, TRPM7, TSFM, TTN, TTR, VCL, XK, ZASP, ZNF365)					
Andersen JD. et al (2019), Denmark [38]	Research article	Whole genome sequencing	Not specified	DNA was purified from whole blood	9 sudden arrhythmic death syndrome (SADS) and 4 sudden unexplained death in infancy (SUDI)	Several genetic variants have been identified in the regulatory regions of heart-associated genes. One of the variants identified was the NEXN variant c.-194A>G, located in the	The study highlights the importance of genetic analysis, particularly analysis of regulatory regions of DNA, in understanding the genetic causes of heart disease that leads to sudden

						promoter region of the NEXN gene. This variant was associated with a significant decrease in NEXN gene expression and cardiac hypertrophy. Significant differences in gene expression levels were observed between cases of sudden arrhythmic death syndrome and cases of sudden unexplained death in infancy. These differences have mainly been associated with cardiac-specific genes and muscle tissue-specific genes.	cardiac deaths, even in individuals with apparently normal hearts at autopsy.
Raju H. et al. (2019), United Kingdom [39]	Retrospective observational study	6 genes (KCNE1, KCNE2, KCNQ1, KCNH2, SCN5A, RYR2)	LQTS, BrS and CPVT	Not specified	197 cases of sudden arrhythmic death syndrome (SADS)	Over the course of the study, rare genetic variants were identified in a total of 29 cases out of 197 patients with sudden arrhythmic death syndrome. Among the rare genetic variants identified,	The study demonstrates the effectiveness of "molecular autopsy" using next-generation sequencing (NGS) to analyze genes associated with cardiac arrhythmia

						10 were classified as pathogenic or probably pathogenic. These mutations were found to be associated with the cardiac arrhythmia syndromes and heart diseases investigated in the study.	syndromes in patients with sudden arrhythmic death syndrome (SADS). This genetic approach can detect genetic mutations associated with these diseases. The NGS sequencing platform demonstrated good sensitivity (100% for rare variants) and specificity (99.99%) in identifying genetic variants. However, it turned out that some variants require confirmation through Sanger sequencing. The study used a specific panel of genes associated with cardiac arrhythmia syndromes. The choice of genes to include in the panel is fundamental to identify the genetic mutations relevant to the pathologies studied. In summary, the study

							provides an overview of the genetic basis of cardiac arrhythmia syndromes in SADS patients and highlights the importance of genetic analysis for the understanding and diagnosis of these pathologies. However, it also highlights the complexity of interpreting genetic variants and the need for further confirmation when identifying rare variants.
Graziosi M. et al. (2020), Italy, [40]	Case report (a 49-year-old man who suddenly died during physical activity)	174 genes (ABCC9, ABCG5, ABCG8, ACTA1, ACTA2, ACTC1, ACTN2, AKAP9, ALMS1, ANK2, ANKRD1, APOA4, APOA5, APOB, APOC2, APOE, BAG3, BRAF, CACNA1C, CACNA2D1, CACNB2, CALM1, CALR3, CASQ2,	Arrhythmogenic left ventricular cardiomyopathy (ALVC)	DNA was isolated from explanted heart paraffin samples		Through genetic testing, two variants of interest were identified in genes associated with arrhythmogenic cardiomyopathy, specifically in the TMEM43 gene and in the DSP gene. A comprehensive cardiac screening was performed in	The study highlighted the importance of the systematic examination of cases of sudden death, with particular attention to autopsies performed by pathologists who are experts in the cardiovascular field and supported by

		CAV3, CBL, CBS, CETP, COL3A1, COL5A1, COL5A2, COX15, CREB3L3, CRELD1, CRYAB, CSRP3, CTF1, DES, DMD, DNAJC19 DOLK, DPP6, DSC2, DSG2, DSP, DTNA, EFEMP2, ELN, EMD, EYA4, FBN1, FBN2, FHL1, FHL2, FKRP, FKTN, FXN, GAA, GATAD1, GCKR, GJA5, GLA, GPD1L, GPIHBP1, HADHA, HCN4, HFE, HRAS, HSPB8, ILK, JAG1, JPH2, JUP, KCNA5, KCND3, KCNE1, KCNE2, KCNE3, KCNH2, KCNJ2, KCNJ5, KCNJ8, KCNQ1, KLF10, KRAS, LAMA2, LAMA4, LAMP2, LDB3, LDLR, LDLRAP1, LMF1, LMNA, LPL, LTBP2, MAP2K1, MAP2K2, MIB1, MURC, MYBPC3, MYH11, MYH6, MYH7, MYL2, MYL3, MYLK,				her 19-year-old son, and after a thorough discussion of the risks and benefits, the son gave consent for implantation of a subcutaneous defibrillator for the prevention of sudden cardiac death.	molecular analyzes to also identify rare hereditary diseases. The identification of specific genetic variants associated with arrhythmogenic left ventricular cardiomyopathy highlights the importance of genetic analysis in understanding inherited cardiac disease. Furthermore, it highlights the need to activate family screening, especially in the presence of cases of sudden death in first-degree relatives, in order to promptly identify any hereditary pathologies and evaluate the opportunity of preventive interventions, such as the implantation of a subcutaneous defibrillator .

		MYLK2, MYO6, MYOZ2, MYPN, NEXN, NKX2-5, NODAL, NOTCH1, NPPA, NRAS, PCSK9, PDLIM3, PKP2, PLN, PRDM16, PRKAG2, PRKAR1A, PTPN11, RAF1, RANGRF, RBM20, RYR1, RYR2, SALL4, SCN1B, SCN2B, SCN3B, SCN4B, SCN5A, SCO2, SDHA, SEPN1, SGCB, SGCD, SGCG, SHOC2, SLC25A4, SLC2A10, SMAD3, SMAD4, SNTA1, SOS1, SREBF2, TAZ, TBX20, TBX3, TBX5, TCAP, TGFB2, TGFB3, TGFBR1, TGFBR2, TMEM43, TMPO, TNNC1, TNNI3, TNNT2, TPM1, TRDN, TRIM63, TRPM4, TTN, TTR, TXNRD2, VCL, ZBTB17, ZHX3, ZIC3)					
Simons E. et al. (2021), Belgium [41]	Case report (a 49-year-old man who died	61 genes (ABCC9, AKAP9, ANK2, CACNA1C,	Long QT syndrome type 1 (LQTS1)	Not specified		The results of the study include the discovery of two	The variant identified in the DSG2 gene was

	suddenly in his sleep)	CACNA2D1, CACNB2, CALM1, CASQ2, CAV3, CTNNA3 DES, DPP6, DSC2, DSG2, DSP, GJA1 (CX43), GJA5 (CX40), GPD1L, HCN4, JUP, KCNA5, KCND3, KCNE1, KCNE2, KCNE3, KCNE5 (KCNE1L), KCNH2, KCNJ2, KCNJ5 (GIRK4), KCNJ8, KCNQ1 (excl. exon 9), LMNA, NKX2-5 (NKX2E), NOS1AP, NPPA, PKP2, PLN, PRKAG2, RANGRF (MOG1), RYR2, SCN1B, SCN2B, SCN3B, SCN4B, SCN5A, SLMAP, SNTA1, TGFB3, TMEM43, TRDN, TRPM4)				genetic variants of uncertain significance, one in the KCNQ1 gene and one in the DSG2 gene, identified through post-mortem genetic analysis. Segregation analysis revealed that the variant in the KCNQ1 gene was inherited from the proband's mother and passed on to her daughter, while the variant in the DSG2 gene was identified as a de novo mutation, not inherited by the proband's children. However, electrophysiological analysis of the KCNQ1 variant showed no significant differences in the electrophysiological function of the ion channel. Furthermore, no relevant pathological	considered the most likely cause of sudden cardiac death in the presented case. There was insufficient evidence to suggest that the variant identified in the KCNQ1 gene represents a significant risk for sudden cardiac death in the proband's relatives. In summary, the study suggests that the variant in the DSG2 gene is the most likely cause of sudden cardiac death in the present case, while the variant in the KCNQ1 gene does not appear to be directly related to the risk of sudden cardiac death in the proband's family.

						cardiac conditions emerged during in-depth cardiac evaluations of the proband's children, despite the presence of the genetic variants. Consequently, attention was placed on the proband's daughter, with the decision to place her on beta-blockers and limit competitive sports participation, as intense physical activity is known to be a potential trigger for arrhythmias in the context of Long QT type 1 (LQT1).	
Gaertner-Rommel A. et al. (2019), Germany [42]	Case report (a 19-year-old male)	174 genes associated with inherited cardiac conditions	HCM	Not specified	DNA was extracted from blood samples	The result of the study indicates that the 19-year-old patient, who died suddenly at school, was diagnosed with hypertrophic cardiomyopathy (HCM) during forensic autopsy. DNA sequencing analysis revealed the	The study highlighted that the patient was a carrier of two pathogenic genetic mutations, one in the MYBPC3 gene and the other in the FHL1 gene, which may have contributed to the appearance of the hypertrophic

						presence of two pathogenic mutations, one in the MYBPC3 gene and the other in the FHL1 gene. The identified FHL1 variant has been classified as a nonsense mutation that causes the complete lack of FHL1 protein. This led to a significant reduction in FHL1 mRNA levels and the absence of the truncated form of the protein in the patient's skeletal muscle tissue and myocardium. On the other hand, the MYBPC3 variant was classified as probable pathogenic according to the ACMG criteria (American College of Medical Genetics). However, the impact of the MYBPC3 variant in the occurrence of the sudden death event	cardiomyopathy that led to his sudden death. The presence of both mutations suggested that the joint effects of both may have caused a more severe cardiac phenotype than either mutation alone. Furthermore, it was observed that the mutation in the FHL1 gene led to a complete absence of the FHL1 protein in the patient's skeletal muscle, suggesting a pathological mechanism of the mutation. The presence of genetic mutations provided important insights to better understand the pathological mechanism of hereditary heart disease in the patient and underlined the importance of genetic analysis in cases of sudden cardiac death.

						was difficult to predict, as it was associated with incomplete penetrance and variable expression variability within a family. These findings suggest that the combined interaction of mutations in the FHL1 and MYBPC3 genes may have contributed to the severity of the patient's cardiac phenotype.	
Mahlke N. et al. (2019), Germany [43]	Case report (a 13-year-old female)	74 genes associated with inherited cardiovascular conditions	Catecholaminergic polymorphic ventricular tachycardia (CPVT))	Not specified		A mutation has been identified in the cardiac ryanodine receptor gene RyR2, which is associated with catecholaminergic polymorphic ventricular tachycardia (CPVT). The specific mutation identified was described as a non-synonymous substitution of the amino acid glycine	The main considerations of the study concern the importance of genetic analysis in cases of sudden death, especially when there is no clear morphological or histological evidence. The authors point out that early identification of genetic mutations is crucial, as it can not

						with glutamic acid (p.Gly2166Glu) due to a heterozygous nucleotide exchange at position 6497 (c.6497G>A) in exon 42 of the RyR2 gene.	only clarify the cause of death in seemingly inexplicable cases, but also help identify affected relatives who may need appropriate treatment to reduce the risk of death sudden heart attack. Furthermore, genetic analysis is considered essential in situations where hereditary cardiovascular conditions are involved, as it can allow the identification of specific mutations that may not be evident during conventional morphological and histological investigations. Finally, the authors highlight the importance of increased awareness among forensic specialists to ensure

							that cases of sudden death are adequately investigated and that potential genetic implications are taken into account for optimal management of at-risk relatives.
Neubauer J. et al. (2019), Switzerland [44]	Case report (a 19-year-old woman)	Whole exome	Not specified	Not specified		In the study described in the provided text, a specific genetic variant, called N1774H, was identified, located in the intracellular C-terminal region of the SCN5A gene. This variant was associated with significant functional and electrophysiological effects, including a 363% increase in late current compared to wild-type channels, as well as a 48% decrease in peak current density. Furthermore, changes in stable activation and	The results obtained suggest that the identified variant has a key role in modulating the activity of sodium channels. Furthermore, the study linked the presence of this genetic variant to a series of clinical manifestations, including QTc prolongation and cases of sudden death in the examined family. This underlines the importance of further investigating the clinical and therapeutic implications of these specific genetic

						inactivation values of sodium channels were observed. This study also identified a number of symptoms and clinical signs related to the variant, including QTc prolongation in several members of the family studied, as well as cases of sudden death. Analysis of family members revealed that the variant was transmitted in a heritable manner to several affected members, causing a variety of clinical and electrophysiological manifestations.	variants, in particular to identify those at risk and to adopt preventive measures such as the implantation of cardiac defibrillators. Finally, protein analysis revealed a significant reduction in expression of the N1774H variant compared to the wild-type protein. This observation suggests further understanding of the underlying pathogenetic mechanisms related to the N1774H variant and may have implications for the development of targeted therapies. However, further research is needed to deepen our understanding of how this variant affects sodium channel function and to develop appropriate

							therapeutic strategies.
Foti F. et al. (2020), Italy [45]	Case report (a 34-year-old man who died suddenly on exercise)	174 genes	Arrhythmic heart disease	DNA was extracted from frozen blood and spleen samples		A rare genetic variant has been identified: a nucleotide substitution in the RyR2 gene that has not been previously reported in the Genomic Aggregation Database (GnomAD). the hypothesis of a de novo mutation in the proband was put forward, since the genetic variant was not found in the parents. A detailed analysis of the RyR2 gene has highlighted the potential role of the genetic variant in compromising the structure and function of cardiac cells, resulting in a possible cause of death.	This study highlights the importance of conducting in-depth investigations, including genetic analyzes and family investigations, when autopsy and toxicological investigations are inconclusive and extracardiac causes are excluded. Furthermore, it highlights the need to involve a multidisciplinary team composed of cardiologists, geneticists, forensic pathologists, and hereditary heart disease specialists for accurate diagnosis and appropriate management of cases of sudden cardiac death. The challenge is to interpret the results of genetic investigations and a fundamental role is

							played by cardiogenetic experts specialized in this evaluation. Finally, we report the importance of further research and studies to fully understand the phenotypic effects and clinical implications of these mutations through the use of in vitro experiments and in-depth assessments of cardiac function.
Manzanilla-Romero HH. Et al. (2023), Austria [46]	Case report (a 15-year-old girl who died in her sleep)	Firstly 48 genes for arrhythmias and then whole exome	Myocarditis	DNA was extracted from blood samples		Two variants of uncertain significance were detected in the SCN5A and CACNA1D genes, followed by the discovery of a specific mutation in the PPA2 gene, associated with a condition of PPA2-related mitochondriopathy. Analysis of family history revealed a possible autosomal	The precise identification of the mutation in the PPA2 gene has been crucial for understanding the disease and adequately treating affected patients. The study highlighted how even small quantities of alcohol can have a significant impact on the cardiac conditions of patients affected by

						dominant genetic transmission with a high risk of cardiac arrest within the family. The importance of an accurate diagnosis is highlighted, as the presence of a precise genetic condition has provided a clear framework for the treatment and management of the disease, in particular the importance of avoiding alcohol intake which could trigger a crash cardiac.	the genetic mutation, underlining the importance of completely avoiding alcohol consumption to avoid cardiac risks. The study also highlighted the importance of conducting detailed and in-depth genetic investigations, especially when initial results are not conclusive. This made it possible to identify the mutation in the PPA2 gene, which paved the way for a better understanding of the condition and more effective management of it. Knowledge of family history was essential to identify a possible hereditary pattern of the condition, thus providing guidance for the diagnosis and treatment of at-risk family members.
